# Eating Disorders and Emotional Intelligence: which relationship?

**DOI:** 10.1192/j.eurpsy.2026.11083

**Published:** 2026-07-31

**Authors:** I. Mannoubi, M. Turki, I. Chaari, M. A. Megdiche, N. Halouani, S. Ellouze, J. Aloulou

**Affiliations:** Psychiatry B, Hedi Chaker hospital, Sfax, Tunisia

## Abstract

**Introduction:**

Eating disorders (ED) represent a major mental health concern and are often associated with emotional and interpersonal difficulties. Emotional intelligence (EI), defined as the ability to perceive, understand, and regulate one’s own emotions as well as those of others, may play a key role in understanding and managing these disorders.

**Objectives:**

This study aimed to examine the relationship between ED and EI.

**Methods:**

We conducted a cross-sectional, descriptive, and analytical study among 161 Tunisian participants via an anonymous online questionnaire (Google Forms). Two validated French instruments were used: the Eating Attitudes Test (EAT-26) to assess ED risk and the Schutte Self-Report Emotional Intelligence Test (SSEIT) to measure EI.

**Results:**

The mean EAT-26 score was 13.60 ± 13.85 (range: 2–75), with 33 participants (20.5%) scoring ≥ 20, indicating a high risk of ED. The mean SSEIT score was 115.52 ± 26.23 (range: 33–165), with 54% of participants (n = 87) displaying average levels of EI. Pearson correlation analysis revealed a significant negative association between EAT-26 and SSEIT scores (r = –0.40; p<0.001), indicating that higher ED risk was associated with lower EI. Furthermore, participants at low risk for ED had significantly higher mean EI scores (119.41) than those at high risk (100.39) (t = 3.07; p=0.0038).

**Conclusions:**

Our findings highlighted a significant association between ED and EI. Lower EI is linked to increased risk of ED, suggesting that interventions targeting emotional skills may be valuable for prevention and management of these disorders.

**Disclosure of Interest:**

None Declared

